# Expression of Concern: Regulation of the breast cancer stem cell phenotype by hypoxia-inducible factors

**DOI:** 10.1042/CS20150451_EOC

**Published:** 2026-09-16

**Authors:** Gregg L. Semenza

**Affiliations:** 1 Institute for Cell Engineering, Johns Hopkins University School of Medicine, Baltimore, MD 21205, U.S.A.; 2 Department of Pediatrics, Johns Hopkins University School of Medicine, Baltimore, MD 21205, U.S.A.; 3 Department of Medicine, Johns Hopkins University School of Medicine, Baltimore, MD 21205, U.S.A.; 4 Department of Oncology, Johns Hopkins University School of Medicine, Baltimore, MD 21205, U.S.A.; 5 Department of Radiation Oncology, Johns Hopkins University School of Medicine, Baltimore, MD 21205, U.S.A.; 6 Department of Biological Chemistry, Johns Hopkins University School of Medicine, Baltimore, MD 21205, U.S.A.; 7 McKusick-Nathans Institute of Genetic Medicine, Johns Hopkins University School of Medicine, Baltimore, MD 21205, U.S.A.

*Clinical Science* has been made aware by a reader of potential issues surrounding the scientific validity of some of the references cited within this paper and is hence issuing a permanent Expression of Concern to notify readers. Seven articles cited have been retracted since the publication of this review, six of which are self-citations. These include the following:

- DOI: 10.1073/pnas.1421438111
- DOI: 10.1073/pnas.1321510111
- DOI: 10.1158/1541-7786.mcr-12-0629
- DOI: 10.1158/0008-5472.can-12-3963
- DOI: 10.1038/onc.2011.365
- DOI: 10.1073/pnas.0909353106
- DOI: 10.1038/nature04695

The author has cooperated with our investigation and provided alternative references that support the topic of this review. As such, the article will remain published but with an associated Expression of Concern.

